# Identification of genic moss SSR markers and a comparative analysis of twenty-four algal and plant gene indices reveal species-specific rather than group-specific characteristics of microsatellites

**DOI:** 10.1186/1471-2229-6-9

**Published:** 2006-05-30

**Authors:** Mark von Stackelberg, Stefan A Rensing, Ralf Reski

**Affiliations:** 1 Plant Biotechnology, Faculty of Biology, Schaenzlestr. 1, 79104 Freiburg, Germany

## Abstract

**Background:**

The moss *Physcomitrella patens *is an emerging model in comparative plant science. At present, the *Physcomitrella *genome is sequenced at the Joint Genome Institute (USA). In this study we present our results on the development of expressed sequence tag-derived microsatellite markers for *Physcomitrella patens*, their classification and applicability as genetic markers on the intra- as well as on the interspecies level. We experienced severe restrictions to compare our results on *Physcomitrella *with earlier studies for other plant species due to varying microsatellite search criteria and a limited selection of analysed species. As a consequence, we performed a side by side analysis of expressed sequence tag-derived microsatellites among 24 plant species covering a broad phylogenetic range and present our results on the observed frequencies.

**Results:**

We identified 3,723 microsatellites using the software MISA in a non-redundant *Physcomitrella *expressed sequence tag database comprising more than 37 megabases of nucleotide information. For 2,951 microsatellites appendant primer sequences have been derived. PCR of 376 microsatellites yielded 88 % successful amplicons and over 30 % polymorphisms between two *Physcomitrella *accessions. The polymorphism information content of 64 microsatellites based on 21 different *Physcomitrella *accessions was comparably high with a mean of 0.47 +/- 0.17. Of the 64 *Physcomitrella *microsatellite markers, 34 % respectively 79.7 % revealed cross-species applicability in two closely related moss species.

In our survey of two green algae, two mosses, a fern, a fern palm, the ginkgo tree, two conifers, ten dicots and five monocots we detected an up to sevenfold variation in the overall frequency with a minimum of 37 up to maximal 258 microsatellites per megabase and a high variability among the different microsatellite class and motif frequencies. Numerous species-specific microsatellite frequencies became evident and several deviations to earlier reports were ascertained.

**Conclusion:**

With the *Physcomitrella *microsatellite marker set a valuable tool has been made available for further genetic and genomic applications on the intra- as well as on the interspecies level. The comparative survey of expressed sequence tag-derived microsatellites among the plant kingdom is well suited for a classification of future studies on plant microsatellites.

## Background

The moss *Physcomitrella patens *(Hedw.) B. S. G. is an important model organism for comparative studies in plant science [1]. The ancestors of mosses and seed plants separated shortly after the transition from water to land at least 500 million years ago [2,3]. The moss *Physcomitrella *is therefore placed in a phylogenetic key position between the green algae and the seed plants. *Physcomitrella *displays an exceptionally high rate of homologous recombination [4], which is a unique characteristic among plants. This facilitates direct replacement of genomic loci to knock-out or knock-in genes in order to enable their fast and straightforward functional characterisation [5]. Functional mutations are furthermore facilitated by the dominating haploid gametophyte of the moss. Besides, *Physcomitrella *is easy to handle *in vitro *and to transfect, and is regarded as a rich source of novel genes [6]. More than 200,000 sequenced cDNA fragments, so called expressed sequence tags (ESTs), derived from the worldwide labstrain 'Gransden' have been assembled and annotated in a non-redundant database, a *Physcomitrella *gene index [7-9]. At present, the *Physcomitrella patens *genome is sequenced by a whole genome shotgun approach at the Joint Genome Institute (USA) and the appendant international moss genome consortium collaborates in processing and assembling the genome data. Little is known about the genome organisation yet. The *Physcomitrella *genome is of intermediate size with about 511 megabases [10] and cytogenetic analyses indicate a chromosome number of *n *= 27 [11]. Neither molecular markers nor genetic linkage maps have been established so far. Thus our objective was to establish EST-derived microsatellites in order to be able to create a genetic map for *Physcomitrella patens*.

Microsatellites or simple sequence repeats (SSRs) denote a DNA class of mono- up to hexanucleotide sequence repeats dispersed over the whole genome with an accumulation in nonrepetitive DNA and untranslated 3'- and 5'-regions of genes [12,13]. SSRs are currently preferentially applied as molecular markers in numerous organisms particularly with regard to their unique hypervariabilty combined with co-dominance, specificity and reproducibility [14,15]. The main disadvantage of SSRs as markers has been their time consuming development in the laboratory [16]. However, with the fast-paced increase of nucleic acid sequences during the last decade it became practicable to screen *in silico *for microsatellites in sequence databases for a growing number of organisms. Several tools have been made available for the computational database mining of SSRs, reviewed in [17]. Apart from genomic sequences, especially the large number of availble ESTs and the respective databases have been used extensively to derive SSRs, for example [18-23]. A big advantage of EST-derived markers is their non-anonymity. Each marker is absolutely linked to a distinct gene and therefore to its known or putative function. Moreover, each marker sequence can be extended by the underlying EST. This in particular can be of great benefit in the implementation of genetic markers and linkage maps as a scaffold for physical mapping.

A clear-cut comparison of the first large scale EST-based SSR search for a moss with results obtained for other plant species was virtually impossible due to the large differences in the applied SSR search strategies concerning the redundancy and the chosen parameters for the minimal SSR motif length or the inclusion/exclusion of imperfect SSRs [17]. In wheat, for example, this differences led to reported SSR frequencies ranging from 57 [24] up to 1,350 [12] SSR per megabase. Furthermore, comparative analyses of SSR frequencies based on large scale computational database searches have been limited to only a few groups of mono- and dicots [12,13,18].

In this study we present our results on the development of SSR markers for *Physcomitrella patens *based on clustered EST data, their classification and characterisation and their applicability as genetic markers on the intra- as well as on the interspecies level. For a better classification of our results on *Physcomitrella *SSRs, we performed a comparative side by side EST-based SSR search in 24 phylogenetically well distributed plant species including two green algae, two mosses, a fern, a fern palm, the ginkgo tree, two conifers, ten dicots and five monocots and present our results on the observed SSR frequencies.

## Results and discussion

### Within the *Physcomitrella patens *gene index dimer SSRs are most frequent

We analysed 48,961 virtual *Physcomitrella *transcripts with the MISA software [25,26]. In 3,108 (6.3 %) of the EST sequences one or more microsatellites were found, 3,723 microsatellite motifs were detected in total. This corresponds to a frequency of 98.8 SSR counts per mega base pairs (counts/Mbp) and one SSR per 10.1 kilo base pairs (kbp). Interestingly, the majority of the SSRs were dimer repeats with 2,095 individual SSRs (56.3 %), whereas only 1,315 (35.3 %) were trinucleotide repeats. This is an unexpected result because trimer repeats are reported to be the most frequent SSRs in plant ESTs [17]. Other SSRs were 273 (7.3 %) tetranucleotides, 39 (1.1 %) pentanucleotides and one hexanucleotide SSR (Table 1). This corresponds to SSR counts/Mbp of 55.6 for dimers, 34.9 for trimers, 7.2 for tetramers, 1.0 for pentamers and 0.03 for hexamers, respectively. Among each of the SSR classes the different possible repeat motifs were not evenly distributed (Table 1). The average motif length including compound SSRs was 21.3 base pairs (bp) with 26 % of the motifs being larger than 20 bp.

472 EST sequences contained more than one SSR. 381 ESTs contained two SSRs (80.7 %), 59 contained three (12.5 %), 23 contained four (4.9 %), six contained five (1.3 %) and one EST each contained six, seven respectively 13 SSRs (each 0.2 %). For the generation of PCR markers, multiple SSRs in one EST being separated by less than 100 bp were defined as in 'compound formation' and subsequently handled as one single potential marker. Multiple SSRs being separated by more than 100 bp were further treated as separate single marker loci. In total, 3,171 SSRs were available as potential marker loci, comprising 2,924 SSRs in single and 247 SSRs in compound formation.

### A high rate of *Physcomitrella *SSRs are PCR applicable

Oligonucleotide primer sequences were successfully derived for 2,951 (93 %) of the 3,171 single and compound microsatellites using the Primer3 [27] software in batch mode with MISA. Primers could not be designed for SSR motifs comprising too short or inappropriately composed flanking sequences.

For 376 SSRs, primer pairs were synthesized and PCR was performed using the standard *Physcomitrella *lab strain 'Gransden'. PCR led to successful product amplifications in 329 of 376 SSRs (87.5 %), of which 27 SSR amplicons (7.2 %) yielded longer sequences than expected (size difference from 0.1 up to 2 kbp), most likely due to presence of introns. The frequency of successfully amplified SSRs was more than 20 % higher than described for *Hordeum vulgare*, where SSR markers were also derived with MISA/Primer3 [25]. This may be due to the high quality of the clustered *Physcomitrella *EST database and our specific PCR conditions with a touchdown PCR and a decreased final primer annealing temperature. Moreover, differing intron frequencies with 22 % in *Hordeum *[25] and 8.4 % in *Physcomitrella*, as detected in SSR amplicons, may also contribute to the higher rate of successful PCRs in *Physcomitrella*.

### The *Physcomitrella *SSR markers display a high degree of polymorphism

**Figure 1:**
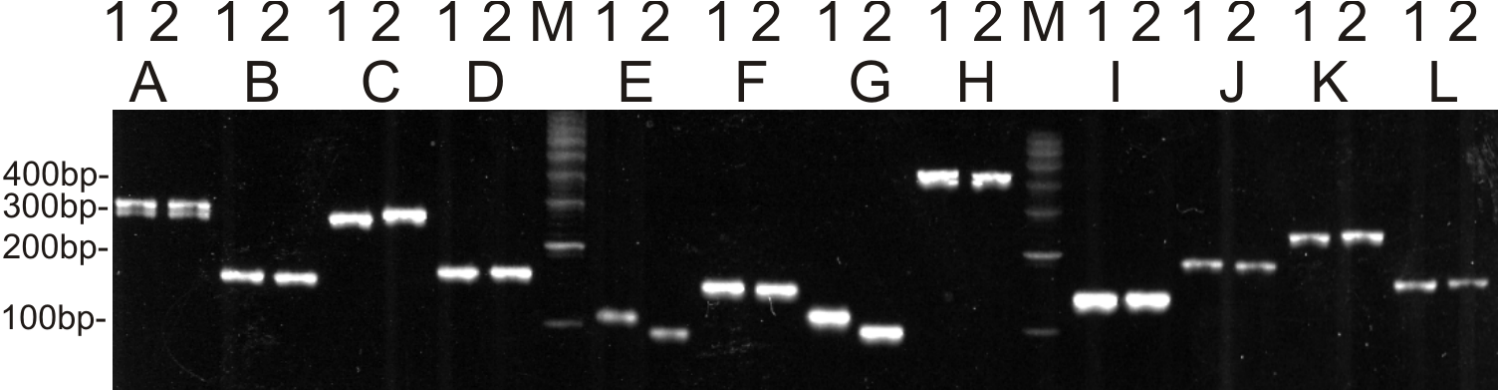
**PCR amplification patterns of EST-derived microsatellites of *Physcomitrella patens *in two accessions**. Exemplary SSR marker A – L were amplified in two *Physcomitrella patens *accessions, the standard lab strain 'Gransden' (1) and 'Villersexel-K3' (2) originating from England and France, respectively. The SSR marker A shows a double banding. The SSRs A, B, D, F and I reveal no visible size polymorphism between the two accessions. The SSRs C, E and G reveal distinct visible single size polymorphisms between the two accessions. The SSRs J, K and L show possible small size polymorphisms between the two accessions which have to be further confirmed. The SSR marker H shows an unexpected size shift, most likely due to an intron. DNA size markers (M) is a 100 bp ladder (SIGMA-Aldrich).

The applicability of the EST-derived SSRs as molecular markers could be proven with side by side amplification of the 376 SSRs in the lab strain 'Gransden' and the french accession 'Villersexel-K3', both of which we are using as parental lines in a genetic mapping approach. 110 markers (33.1 %) were polymorphic between the two accessions (Figure 1).

**Figure 2:**
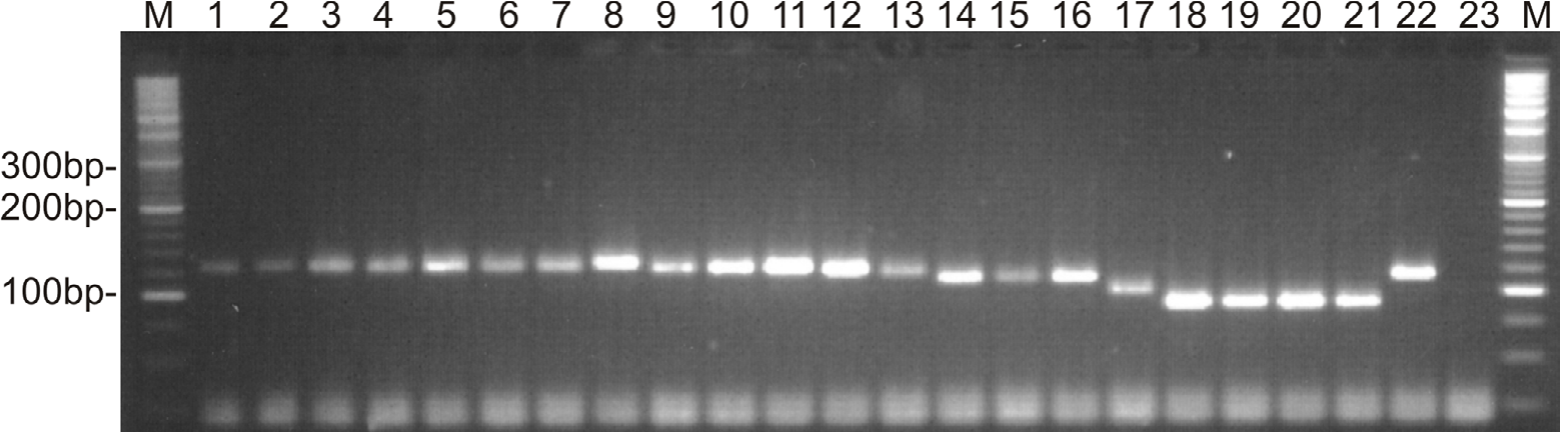
**SSR allele frequency among 21 *Physcomitrella patens *accesssions and two related *Funariaceae***. One exemplary SSR marker was amplified in 21 *Physcomitrella patens *accessions (1, Gransden; 2, NeneWashes; 3, Cholsey; 4, Gemuend-K1; 5, Gemuend-K5; 6, Honnef; 7, Ukraine; 8, Trondheim; 9, Kaskaskia; 10, DelValleLake; 11, Villersexel-K3; 12, Villersexel-K4; 13, Uppsala; 14, Heimerbruehl; 15, Nennig; 16, Martinshof; 17, Bisoke; 18, Okayama; 19, Kumamoto; 20, Saitama; 21, Victoria;) and two related *Funariaceae*, *Physcomitrium sphaericum *(22) and *Funaria hygrometrica *(23). The SSR marker reveals at least six distinct alleles. Single alleles were found in the plants 1 – 13, 14 – 16, 17, 18 – 21, 22 and 23 respectively. Among the plants 1 – 13 potentially more alleles are present with only small size differences. DNA size markers (M) are a combination of a 100 bp and a 20 bp ladder (SIGMA-Aldrich).

The informative properties of the EST-derived microsatellites were further evaluated in 64 SSR markers selected from the 376 markers and a collection of 21 worldwide *Physcomitrella *accessions (Table 2, Figure 2). In total, 238 alleles, including 30 null alleles (13.2%), were detected with a maximum of 7 alleles and an average of 3.7 alleles per SSR. Only one SSR turned out to be monomorphic in the analysed accessions. The polymorphism information content (PIC) based on the 21 *Physcomitrella *accessions was calculated for all 64 SSR markers. PIC values ranged from 0.0 – 0.78 with a mean PIC value of 0.47 ± 0.17. This PIC is comparably high for EST-derived SSRs, which are generally known to be less informative than SSRs derived from genomic sequences [28,29]. An explanation for the high amount of SSR polymorphism, including numerous null alleles, could be a high degree of genetic diversity in the studied accessions.

### *Physcomitrella *SSR markers are cross-species applicable

*Physcomitrella patens *belongs to the family *Funariaceae*. To gain insight into the interspecies transferability, the 64 *Physcomitrella *SSR marker were analysed in two further species of the *Funariaceae*, *Physcomitrium sphaericum *and *Funaria hygrometrica *(Table 1, Figure 2). Whereas 51 SSR PCRs (79.7 %) performed well in the more closely related *Physcomitrium sphaericum*, only 22 (34 %) did so in the more distantly related *Funaria hygrometrica *(Figure 2). Given this data, we estimate that for *Physcomitrium sphaericum *about 2,350 *Physcomitrella *SSRs with the appendant primer pairs can be transferred directly and still about 1,000 *Physcomitrella *SSRs are applicable in *Funaria hygrometrica*. Hence, researchers working on closely related moss species may benefit from the *Physcomitrella *SSRs.

Our results are in accordance with prior reports about interspecies transferability of EST-derived SSRs for numerous seed plants including *Triticum aestivum*, *Hordeum vulgare*, *Festuca arundinacea*, *Oryza sativa*, *Medicago truncatula *and *Picea taeda*, where the transferability decreased with increasing phylogenetic distance and transfer success rates differed from 96 % to 40 % [17,25,30-32].

### Datasets of 24 plant species covering a broad phylogenetic spectrum

We aimed to achieve a clear-cut comparison of our results for *Physcomitrella *with those of other plant species. Unfortunately, to classify surveys on EST-derived SSRs was rather complicated due to large differences in the applied SSR search strategies and by the limited number of analysed species. As a consequence, we identified microsatellites in a comparative side by side search using the MISA software upon the gene indices of 24 plants species. Accounting for the present availability of EST sequences, the species selection encompasses as many major clades of the plant kingdom as possible. In total, two green algae, two true mosses, a fern, a fern palm, the ginkgo tree, two conifers, ten dicots and five monocots were analysed (see Additional file 5). However, for some classes among the plants no appropriate EST datasets were available, e. g. liverworts, hornworts and magnoliidae.

The chosen plant sequence datasets consisted of assembled and non redundant EST sequences ('tentative consensus sequences') and of single non redundant ESTs ('singletons'). While the gene indices were build using the same principal method, there are slight variations in the chosen assembly parameters for each of the four databases they originate from, namely The Institute for Genomic Research (TIGR) [33,34], Plant Genome Database (PlantGDB) [35,36], COSMOSS [9,37] and New York Plant Genomics Consortium (NYPG) [38-40]. Methodological variations in generation of the underlying cDNA sequences, e. g. 5'-, 3'- or full length cDNA enrichment, may have led to additional bias in the datasets. Since EST databases are available for only a limited number of species yet, and because they may be affected by a certain bias, care has to be taken in relating deviating observations to certain plant classes or biological characteristics.

The sequence databases ranged in size from 2 megabases up to over 93 megabases of nucleotide information. The overall average EST sequence length was 733 bp with a standard deviation of 180 bp (see Additional file 5). Significantly deviating sequence lengths were detected for *Aquilegia*, *Arabidopsis *and *Oryza *with long average sequence lengths of 1129, 1184 and 1053 bp and *Adiantum*, *Cycas *and *Helianthus *with short average sequence lengths of 483, 476 and 478 bp, respectively. The long sequence averages indicate a better coverage, whereas the short ones most likely are due to a more fragmentary coverage of the full length cDNAs. The dependency of the long average sequence length on coverage of the individual transcript could be confirmed for the *Arabidopdsis *GI which contains more than 5,000 full length cDNA sequences [33]. Because of the extreme EST sequence length variablity in the different datasets, we believe it to be better to compare EST-derived SSR frequencies by calculating the SSR counts per megabase (counts/Mbp) rather than by SSRs per EST as has sometimes been done previously [19,20,30].

The average GC-content in the 24 datasets was 45.4 % with a standard deviation of 5.3 %. Significantly increased GC-contents were detected for the green algae *Chlamydomonas *and *Mesostigma *with 58.3 % and 51.7 %, respectively, the moss *Tortula *with 53.0 % as well as the grasses (51.1% for *Triticum*, 52.5 % for *Hordeum*, 51.5 % for *Saccharum *and 53.7 % for *Oryza*). *Ginkgo*, *Aquilegia *and *Medicago *deviated from the average with significantly reduced GC-contents of 40.0 %, 40.0 % and 39.4 % respectively.

### The overall SSR frequency varies up to sevenfold among plants

**Figure 3:**
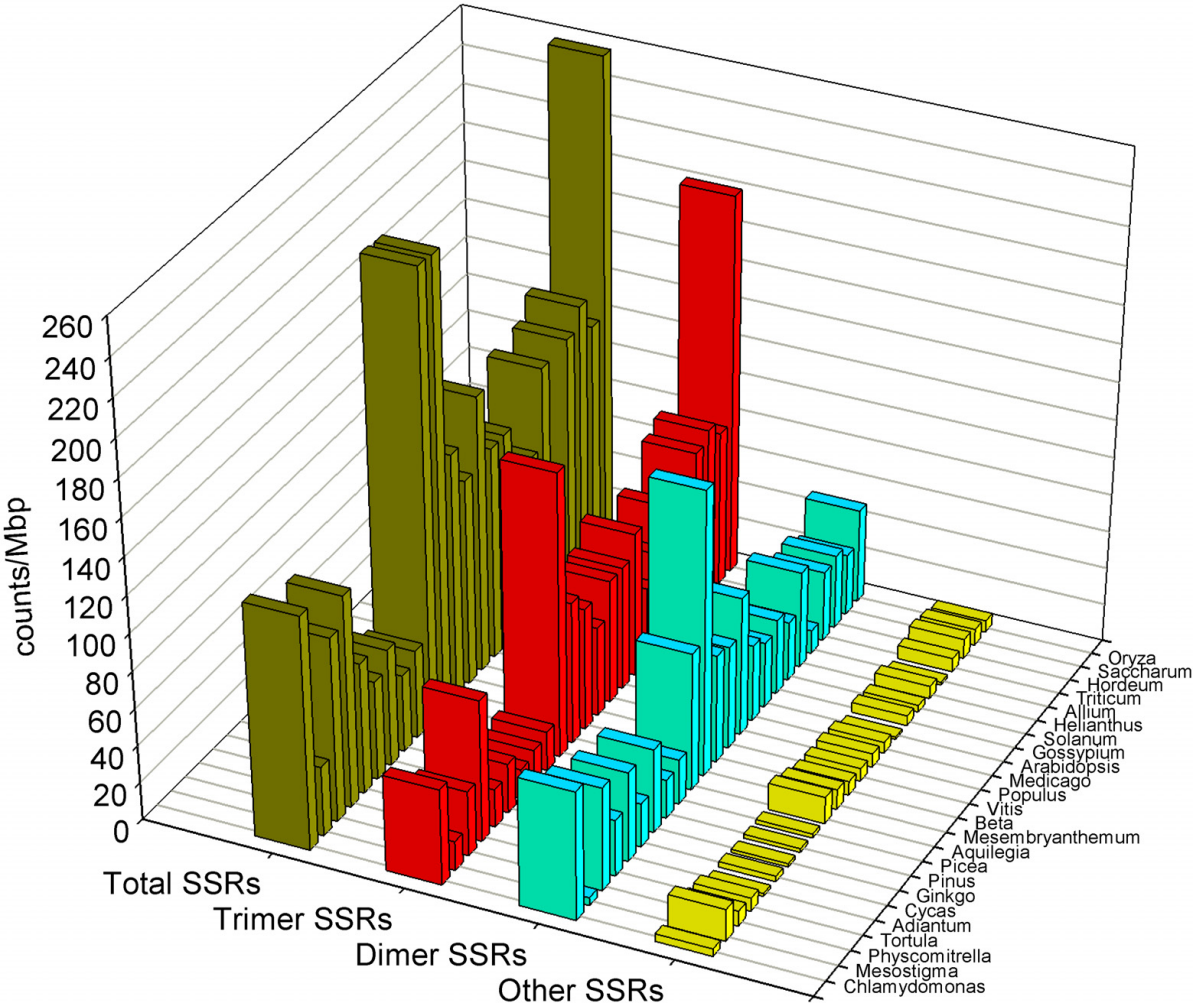
**Frequencies of microsatellites among the gene indices of 24 plants**. The microsatellite frequencies are calculated in SSR counts per megabase nucleotide information (counts/Mbp) as detected in the gene indices of 24 phylogenetically well distributed plant species. The total SSR frequency is further subclassified into trimer, dimer and other SSRs. Other SSRs consist of tetramer, pentamer and hexamer SSRs (see also Additional file 1).

The MISA search statistics for all 24 analysed plant species have been made available at COSMOSS [41]. The overall frequency of SSRs varied 6.9 fold among the 24 gene indices (Figure 3, see also Additional file 1). The average SSR frequency was 114.7 counts/Mbp with a standard deviation of +/- 60.1 counts/Mbp. The green alga *Mesostigma*, the fern palm *Cycas*, both conifers (*Pinus *and *Picea*) as well as the monocot *Allium cepa *were significantly deviating from the average with an extremely reduced SSR frequency of 37.3, 52.9, 41.5, 47.3 and 38.9 counts/Mbp, respectively. The monocot *Oryza*, however, as well as the dicots *Aquilegia *and *Mesembryanthemum *revealed a significantly increased frequency with 258, 240 and 239 counts/Mbp, respectively. The high SSR frequency of rice has been reported in earlier results [18]. The hitherto unnoted significantly biased frequencies of the further seven species indicate a much higher variability of EST-derived SSR frequencies among plants than previously reported. Our data for *Cycas*, *Ginkgo*, *Picea *and *Pinus *seem to indicate that a low SSR frequency is an intrinsic characteristic for gymnosperm gene indices. Among the monocots we ascertained the highest variability in the SSR frequencies with *Allium *(38.9 counts/Mbp) and *Oryza *(258.0 counts/Mbp).

Pearson's correlation coefficients (R) of the SSR frequencies with the average GC-content of the analysed species were not significant with -0.12 for dimer SSRs, 0.23 for trimer, 0.24 for other SSRs and 0.04 for the total SSRs. Thus correlations of SSR frequencies with the average GC-content of the analysed species could not be concluded based on our data.

### Trimer and dimer SSRs are most frequent except for the alga *Mesostigma*

In all datasets, more than 91 % of the detected SSRs were dimer and trimer repeats with the exception of the green alga *Mesostigma *where they accounted for only 58 % of the total SSRs. Among tetra-, penta- and hexamers, the tetramers are the most abundant in 20 of 24 plants, whereas in the remaining four plant species hexamer SSRs are most abundant (Figure 3, see also Additional file 1).

The ratio of the two most common SSR classes, the dimer and trimer repeats, varied among the datasets. In 15 plants trimer SSRs were more frequent than dimer SSRs. Interestingly, the higher dimer than trimer SSR frequency of *Physcomitrella *was also true for eight more species among the analysed sample. This dominance of dimer SSRs in nine of 24 plants contradicts the general assumption that trimer SSRs are most frequent in plant ESTs [17].

The average frequency of trimer SSRs is 61.5 counts/Mbp in the 24 datasets with a standard deviation of +/- 42.8 counts/Mbp. *Mesostigma*, *Ginkgo*, and *Pinus *deviate significantly with only 15.7, 15.8 and 18.6 counts/Mbp, respectively. *Aquilegia *and *Oryza *deviate significantly with 151.8 and 201.8 counts/Mbp. The extreme dominance of trimer SSRs is the main contribution to the observed overall SSR increase in *Aquilegia *and even more evidently in *Oryza*.

The average frequency of dimer SSRs is 46.7 counts/Mbp with a standard deviation of +/- 28.4 counts/Mbp. *Mesostigma*, *Allium *and *Mesembryanthemum *deviate significantly from the average with only 4.3, 12.6 and remarkable 152.4 counts/Mbp, respectively. The observed significant overall SSR increase in *Mesembryanthemum *is due to its extremely high dimer SSR frequency. In *Mesostigma *and *Allium*, significantly reduced trimer as well as dimer SSR frequencies were the reason for the low overall SSR frequency.

In *Mesostigma*, the observed frequencies were especially remarkable (see Additional file 1 and the *Mesostigma*-MISA statistics on COSMOSS [41]): Although it showed the lowest overall SSR frequency of all analysed plants it revealed by far the highest tetramer SSR frequency with 14.1 counts/Mbp (37.9 % of the total SSRs) and furthermore the second highest pentamer SSR frequency with 2.9 counts/Mbp (7.9 % of the total SSRs). The increased tetramer frequency was mainly due to an increased AATT/TTAA motif frequency (9.9 counts/Mbp) and the increased pentamer frequency due to an increased AAATT/AATTT frequency (2.4 counts/Mbp).

### Dimer SSRs averagely are longer and more variable in length than trimer SSRs

**Figure 4:**
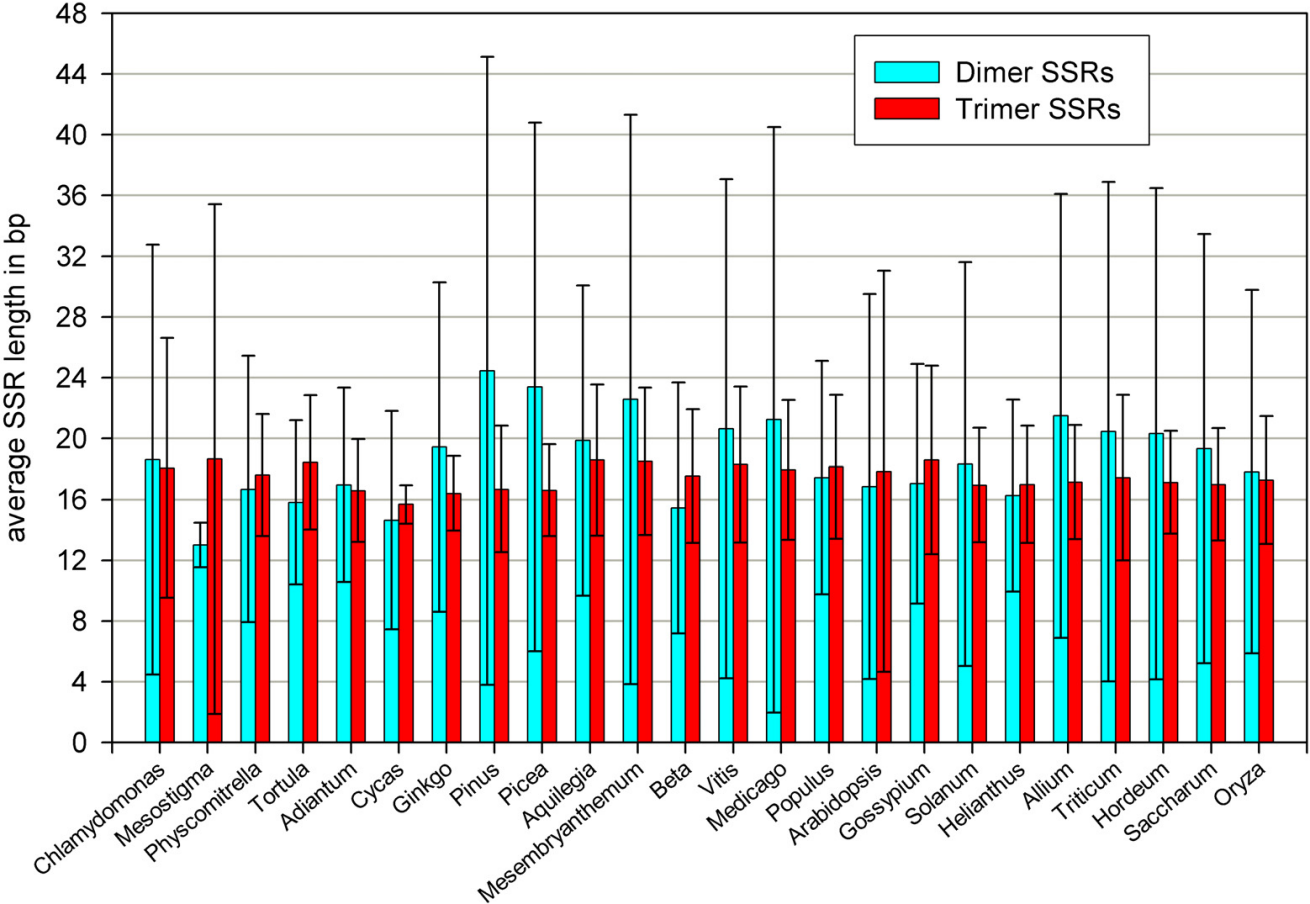
**Average length of dimer as well as trimer microsatellites among the gene indices of 24 plants**. The average microsatellite length of dimer as well as trimer SSRs in basepairs (+/- standard deviation) as detected in the gene indices of 24 phylogenetically well distributed plant species (see also Additional file 2).

Dimer SSRs are much more variable in length throughout the analysed species than trimer SSRs with the exception of *Mesostigma *(Figure 4, see also Additional file 2). The average standard deviation of the dimer SSR length with +/-11.9 bp is more than two times larger than that of trimer SSRs with only +/- 5.2 bp. On average, dimer SSRs (18.7 bp +/- 2.9) were significantly longer (p = 0.03) than trimer SSRs (17.5 bp +/- 0.8). The reduced average dimer length in *Mesostigma *of only 13.0 bp with a small standard deviation (+/- 1.4 bp) might be correlated to the corresponding reduced dimer counts/Mbp. The average dimer length was longest in the two conifers with 24.5 bp, respectively 23.4 bp. Further significant deviations from the average SSR length (see Additional file 2) could neither be correlated with SSR counts/Mbp nor with certain taxonomic clades.

### The SSR dimer motif AG/CT is exceptionally abundant in *Mesembryanthemum*

**Figure 5:**
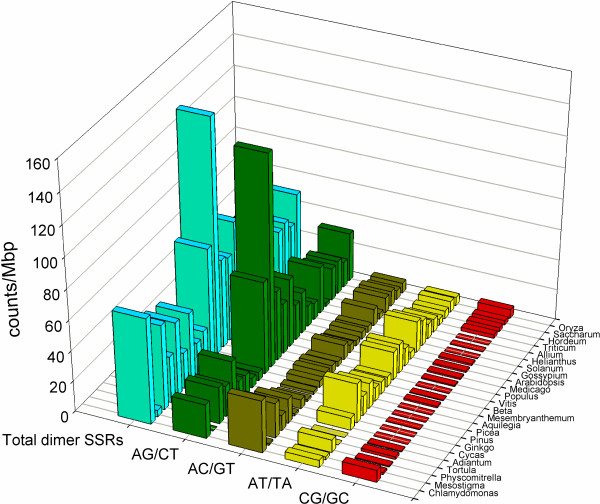
**Frequencies of dimer microsatellite motifs among the gene indices of 24 plants**. The microsatellite frequencies are calculated in SSR counts per megabase nucleotide information (counts/Mbp) as detected in the gene indices of 24 phylogenetically well distributed plant species. The total dimer SSR frequency is further subclassified into the four canonical non-redundant motifs (see also Additional file 3).

The abundancies of the four canonical non-redundant SSR dimer motifs varied strongly among the analysed plants (Figure 5, see also Additional file 3). The average counts/Mbp for the SSRs motifs AG/CT, AC/GT, AT/TA and CG/GC were 27.4 (+/- 27.6), 7.8 (+/- 7.5), 9.8 (+/- 6.8) and 1.3 (+/- 1.9). The large standard deviations reflect the extremely varying motif abundancies in the analysed species. As an amendment to earlier studies, where AG/CT was found to be the most abundant dimer motif in several plants [12], in our analysis this was persistent for only 16 of the 24 datasets. In *Chlamydomonas*, AC/GT and in *Mesostigma*, *Ginkgo*, *Picea*, *Pinus*, *Gossypium*, *Solanum *and *Allium *AT/TA was the most abundant dimer SSR motif. For *Picea*, the high abundance of AT/TA repeats has been noted in earlier reports [42]. In *Mesembryanthemum *an exceptionally high frequency of the AG/CT motif with 140 counts/Mbp is the main contribution to the species increased overall SSR frequency.

### Observed abundancies of SSR trimer motifs amend earlier reports

**Figure 6:**
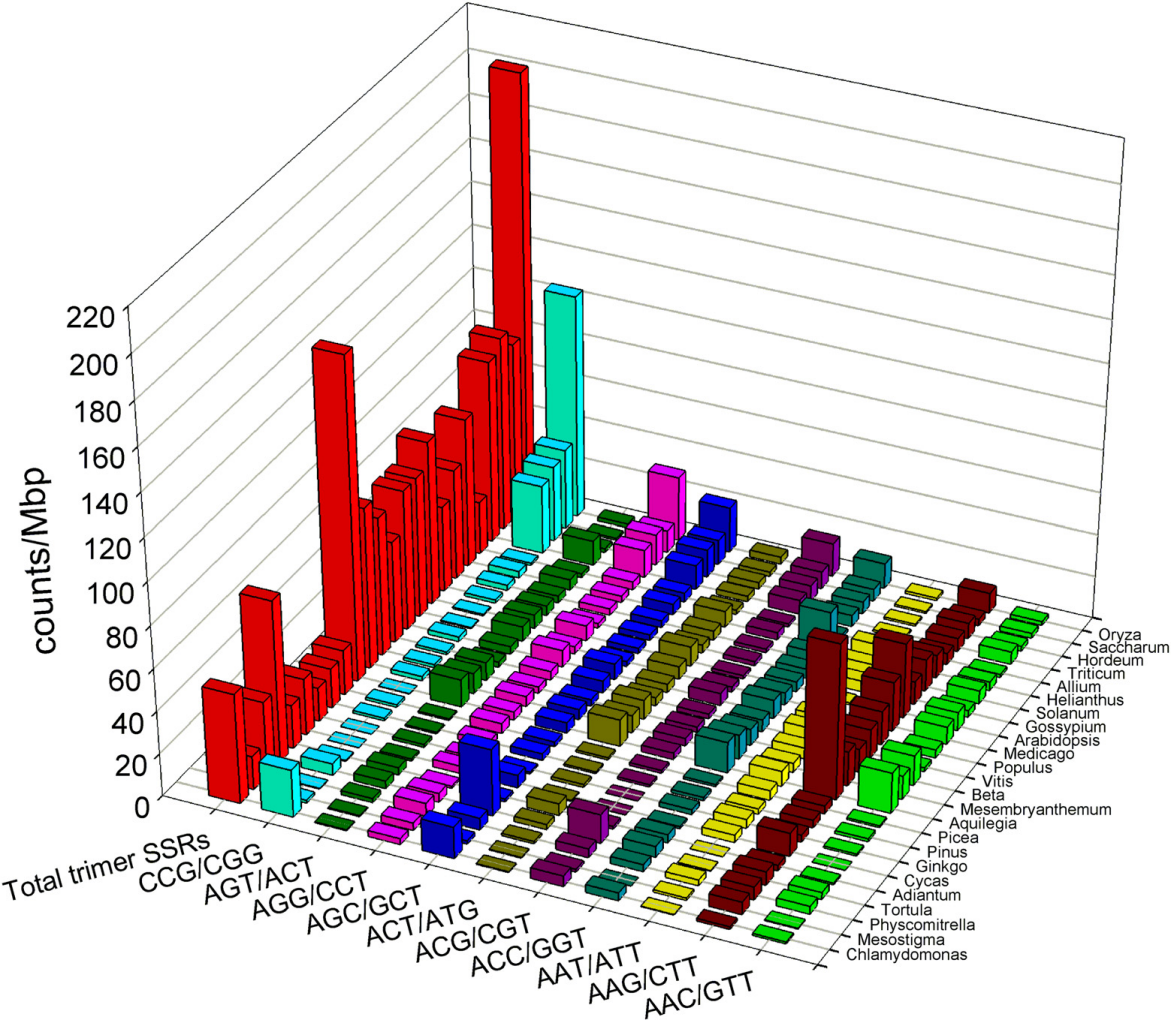
**Frequencies of trimer microsatellite motifs among the gene indices of 24 plants**. The microsatellite frequencies are calculated in SSR counts per megabase nucleotide information (counts/Mbp) as detected in the gene indices of 24 phylogenetically well distributed plant species. The total trimer SSR frequency is further subclassified into the ten canonical non-redundant motifs (see also Additional file 4).

The abundancies of the ten canonical non-redundant SSR trimer motifs varied as well (Figure 6, see also Additional file 4). The average counts/Mbp for the SSRs motifs were 10.8 (+/- 23.3) for CCG/CGG, 4.0 (+/- 3.6) for AGT/ACT, 6.0 (+/- 6.2) for AGG/CCT, 7.1 (+/- 7.2) for AGC/GCT, 4.0 (+/- 3.3) for ACT/ATG, 4.0 (+/- 4.0) for ACG/CGT, 5.8 (+/- 4.6) for ACC/GGT, 3.5 (+/- 2.8) for AAT/ATT, 12.5 (+/- 15.0) for AAG/CTT and 4.4 (+/- 4.8) for AAC/GTT. Like for the dimer motifs, the large standard deviations reflect the extremely varying trimer motif abundancies in the analysed species. In the four grasses, CCG/CGG was the most abundant trimer motif and the same was due for *Chlamydomonas*. The increased CCG/CGG frequency has been described earlier for grasses and has been related to a high general GC-content [12]. In this context the CCG/CGG increase in *Chlamydomonas*, which has the the highest GC-content (58.3 %) of all 24 gene indices, was consistent, whereas the low CCG/CGG frequency in the alga *Mesostigma *and the moss *Tortula*, which also had significantly increased GC-contents of 51.7 and 53.0 %, respectively, did not follow the earlier assumed rule.

The detected low CCG/CGG frequency in *Allium *contradicts the earlier reported generalisation that an increased CCG/CGG frequency is specific for monocots [12,18,43]. An exeptionally high frequency of the SSR motif AAG/CTT contributes to the increased overall SSR frequency in *Aquilegia*. The canonical motifs encompassing the three stop codons AAT, ATG and AGT were not exceptionally reduced most likely due to the presence of the untranslated regions in the EST databases.

## Conclusion

In this study we present the first comprehensive overview of EST-derived microsatellites in a moss, the model plant *Physcomitrella patens*. We detected 3,723 SSRs *in silico *using clustered and assembled EST data. All 2,951 derived primer sets are publicly available, making the markers accessible for PCR analysis. 88 % of the SSRs can be successfully applied to PCR. With the EST-derived SSR marker set a valuable tool has been made available for numerous further genetic and genomic applications on the intra- as well as on the interspecies level. Using the SSR markers a genetic map can be established, the available sequence extensions by the underlying ESTs will greatly facilitate the implementation of the SSR marker loci during the pending iterative assembly process of the *Physcomitrella *genome sequence data.

In our comparative survey microsatellites were found with highly variable abundancies within the EST datasets of 24 phylogenetically well distributed plant species. Interestingly, species belonging to the same phylogenetic group, such as the two mosses, the dicots as well as the monocots did not reveal consistent class-specific SSR characteristics. Rather, species-specificities seem to contribute to the high overall variation. Two exceptions to this rule are the low overall SSR frequency of gymnosperm ESTs, which may be an intrinsic characteristic, and the abundance of the SSR trimer motif CGG/CCG, which seems to be a distinct feature of grasses.

The earlier assumed general dominance of trimer SSRs in ESTs was true for only 15 of the 24 analysed plant species. Dimer SSRs on average were longer and revealed a much higher variability in length than trimer SSRs. Correlations of SSR frequencies with the average GC-content could not be concluded. While the previously reported connection of a high general GC-content to an increased frequency of the trimer SSR motif CGG/CCG was valid for four grasses and *Chlamydomonas*, this was not true for the alga *Mesostigma *and the moss *Tortula*. The earlier assumed general high abundance of the SSR motif CCG/CCG for monocots was not true for *Allium*. Our survey is well suited for a classification of future studies on EST-derived SSRs.

## Methods

### Plant material

A collection of 21 worldwide *Physcomitrella *accessions including the standard laboratory accession 'Gransden' plus two related *Funariaceae*, *Physcomitrium sphaericum *and *Funaria hygrometrica*, were selected for analysis (Table 1). The collection includes japanese, australian, african, european and american accessions [44]. Details of the collection will be presented elsewhere (Mark von Stackelberg Gabriele Schween, Stefan A. Rensing, Ralf Reski, manuscript in preparation). Plants were grown axenically on solid media and in liquid culture according to the small scale cultivation protocol as described [45]. Prior to DNA extraction, plant material was harvested, shock frozen in liquid nitrogen and stored at -80°C.

### DNA extraction

The DNA extraction protocol is based on Doyle and Doyle [46]. Approximately 0.3 g plant material was ground in liquid nitrogen to a fine powder and incubated in 5 mL prewarmed CTAB buffer (2% CTAB, 1.4 M NaCl, 20 mM EDTA, 0.5 % PVP 40, 100 mM Tris [pH 8.0], 0.2 % [v/v] beta-mercaptoethanol) at 65°C for one hour with occasional agitation in a 15 mL polypropylene tube. Afterwards, 5 mL chloroform : isoamylalcohol (24 : 1) was added. Organic and aqeous phase were vigorously mixed followed by separation at 2,500 g for 10 minutes. The aqeous phase was then transferred into a fresh 15 mL polypropylene tube. RNAse A was added to a final concentration of 100 μg/mL and the solution incubated for 30 minutes at 37°C. One tenth volume sodium acetate (3 M, pH 5.2) was added and DNA precipitated overnight with one volume isopropanol at -20°C. DNA was pelleted for 30 min at 2,500 g/4°C. The supernatant was decanted and the pellet incubated in 10 mL washing solution (76 % ethanol, 10 mM ammoniumacetate) for 20 minutes at room temperature (RT). After a short centrifugation, washing buffer was decanted and 70 % ethanol added for a 5 minute incubation at room temperature. After a short spin the supernatant was removed completely and the pellet air dried at room temperature. DNA was dissolved in 200 μL TE buffer overnight at 4°C. The solution was warmed to 65°C for 10 minutes to promote dissolving. DNA quality and concentration was examined by electrophoresis in 0.8 % agarose gels.

### Sequence datasets

For a comparative microsatellite analysis, clustered EST sequence datasets of 24 plant species were chosen (see Additional file 5). For 18 species we downloaded the assembled non-redundant EST-based nucleotide sequence datasets (tentative contigs of the gene indices) from TIGR [33,34] and for two species each from PlantGDB [35,36] and from NYPG [38-40]. For the moss *Physcomitrella patens *we used a non-redundant gene index of the standard lab strain 'Gransden' consisting of high quality, vector-clipped, clustered, assembled and annotated ESTs available via COSMOSS [9,37]. The same filtering and assembly procedure as for *Physcomitrella *was applied to derive a gene index for all available EST sequences of the moss *Tortula ruralis*, also available via COSMOSS. All applied databases are summarized in See Additional file 5. The percentage of the nucleotides guanin and cytosin (GC-content) was calculated for the gene indices with the EMBOSS [47] program GEECEE.

### SSR mining software

SSR motifs were detected using the Perl script MISA [25,26]. The MISA definition of microsatellites was by unit size (x) and minimum number of repeats (y): 1/100, 2/6, 3/5, 4/5, 5/5, 6/5 (x/y). Mononucleotides were excluded from further analysis. The maximal number of interrupting basepairs in a compound microsatellite was set to 100. MISA detects perfect SSRs only.

### Analysis of *Physcomitrella *EST-derived microsatellites

For *Physcomitrella patens*, MISA was used in direct conjunction with the Primer3 software [27] to derive appropriate PCR oligonucleotides. The results of the microsatellite search for *Physcomitrella patens *are available at COSMOSS [41]. A maximum of three different primer pairs per SSR have been provided. Furthermore PCR results for 376 microsatellites, polymorphic SSRs between 'Gransden' and 'Villersexel-K3' and PIC values of 64 SSRs for 21 *Physcomitrella *accessions have been provided at COSMOSS [41].

The length of the SSR PCR amplicons was set to 100 – 280 bp. Oligonucleotide parameters for Primer3 were set to a length of 18 – 27 bp with an optimum of 20 bp, a GC content of 20 – 80 % with an optimum of 50 %, a melting temperature (Tm) of 57 – 63°C with an optimum of 60°C, and a primer Tm maximum difference of 1°C. SSR marker were amplified in a 20 μL PCR mix containing 2 μL of 10 × RED-Taq-PCR buffer, 0.1 mM dATP, dCTP, dGTP and dTTP, 5 pmol each of two primers, 0.5 Units RED-Taq-Polymerase (SIGMA-Aldrich) and 4 ng plant DNA. Cycling was carried out in a Biometra thermal cycler T1 starting with an initial DNA denaturation at 95°C for 2 min. The first cycle consisted of 30 sec denaturation at 92°C, primer annealing for 30 sec at 60°C and elongation for 30 sec at 72°C. In each of the 10 subsequent cycles the annealing temperature was decreased by 0.7°C. The final 25 cycles consisted of 15 sec denaturation at 92°C, 15 sec primer annealing at 52°C and 30 sec elongation at 72°C.

Electrophoretic size separation of SSR PCR products was performed in 3 % MetaPhor (Cambrex) high resolution agarose gels in 0.5 fold TBE (45 mM Tris-borate, 1 mM EDTA, pH 8.0). We thereby circumvented the lengthy and elaborate use of denaturing polyacrylamide gels. As described in [48] MetaPhor agarose is capable of resolving microsatellite size differences of only two basepairs. The comparably high costs of this agarose can be reduced by reusing it several times.

### SSR-PCR evaluation

SSRs were scored visually in the gel according to their amplified fragment size. Different sizes of one marker were scored as different alleles. Same sizes of one SSR and different sizes which could not be visually distinguished were scored as the same allele. Repeated abscence of PCR products in 'Gransden'were scored as PCR failures. Repeated absence of PCR products in the 20 additional accessions were scored as null alleles if PCR worked out in 'Gransden'. The polymorphism information content (PIC value) was calculated according to Botstein et al. [49] as follows:

PIC=1−∑j=1nPij2
 MathType@MTEF@5@5@+=feaafiart1ev1aaatCvAUfKttLearuWrP9MDH5MBPbIqV92AaeXatLxBI9gBaebbnrfifHhDYfgasaacH8akY=wiFfYdH8Gipec8Eeeu0xXdbba9frFj0=OqFfea0dXdd9vqai=hGuQ8kuc9pgc9s8qqaq=dirpe0xb9q8qiLsFr0=vr0=vr0dc8meaabaqaciaacaGaaeqabaqabeGadaaakeaacqqGqbaucqqGjbqscqqGdbWqcqGH9aqpcqaIXaqmcqGHsisldaaeWbqaaiabdcfaqjabdMgaPjabdQgaQjabikdaYaWcbaGaemOAaOMaeyypa0JaeGymaedabaGaemOBa4ganiabggHiLdaaaa@3EA9@

where *Pij *is the frequency of the *j*th allele for marker *i *and summation extends over *n *alleles.

## Authors' contributions

MvST conceived of this project, carried out the wet-lab analyses of *Physcomitrella patens *SSRs, performed the computational analyses and drafted the manuscript. SAR implemented the sequence analysis tools and helped with drafting the manuscript. RR helped with conceptualizing the study and with drafting the manuscript.

## Supplementary Material

Additional file 5 **PDF file with a table of the analysed plant gene indices**. Tabular summary of the analysed plant gene indices including plant species name, taxonomic clade, source of database, release date, release version if specified in brackets, total number of sequences, total size of the databases in basepairs, average sequence length in basepairs and the GC-content of the databases. Click here for file

Additional file 1 **PDF file with the original data used to prepare the diagram in **Figure 3. The additional file 1 contains the sizes of the analysed gene indices in bp, the total counts of detected SSRs, the total counts of dimer, trimer, tetramer, pentamer and hexamer SSRs as well as the calculated SSR counts per megabase for dimer, trimer, tetramer, pentamer and hexamer repeats. Click here for file

Additional file 2 **PDF file with the original data used to prepare the diagram in **Figure 4. The additional file 2 contains the average length of dimer as well as trimer SSRs in the analysed 24 plant gene indices with the standard deviation in bp. Click here for file

Additional file 3 **PDF file with the original data used to prepare the diagram in **Figure 5. The additional file 3 contains the sizes of the analysed gene indices in bp, the total dimer SSR counts, the counts of the four canonical dimer SSR motifs as well as their calculated counts per megabase. Click here for file

Additional file 4 **PDF file with the original data used to prepare the diagram in **Figure 6. The additional file 4 contains the sizes of the analysed gene indices in bp, the total trimer SSR counts, the counts of the 10 canonical dimer SSR motifs as well as their calculated counts per megabase. Click here for file

## Figures and Tables

**Table 1:** Frequencies of non-redundant SSR motifs with respect to repeat numbers and *in total *as detected with the software MISA in the *Physcomitrella patens *gene index.

SSR Motif	Number of repeats											Total
		
	5	6	7	8	9	10	11	12	13	14	≥15	
AC/GT	n. d.	461	191	70	60	36	18	8	5	6	8	863
AG/CT	n. d.	308	150	99	59	38	41	39	38	43	88	903
AT/AT	n. d.	80	52	40	41	42	17	12	7	4	12	307
CG/CG	n. d.	19	2	1	-	-	-	-	-	-	-	22
AAC/GTT	72	27	17	14	6	1	-	1	-	-	-	138
AAG/CTT	104	42	28	24	23	3	4	-	1	-	-	229
AAT/ATT	34	24	18	11	7	4	3	-	1	-	-	102
ACC/GGT	76	38	9	-	-	-	-	-	-	-	-	123
ACG/CTG	102	33	13	8	1	2	-	-	-	1	-	160
ACT/ATG	24	8	3	-	5	1	-	-	-	-	-	41
AGC/CGT	123	52	18	11	3	1	2	-	-	-	1	211
AGG/CCT	144	46	27	9	2	-	-	-	-	-	-	228
AGT/ATC	51	12	2	3	1	-	1	-	-	1	-	71
CCG/CGG	8	1	2	1	-	-	-	-	-	-	-	12
AAAC/GTTT	16	11	5	1	2	-	-	-	-	-	-	35
AAAG/CTTT	8	1	-	-	-	-	1	-	-	-	-	10
AAAT/ATTT	40	18	5	-	-	-	-	-	1	-	-	64
AAGG/CCTT	5	2	2	2	1	-	-	-	-	-	-	12
ACAG/CTGT	4	5	-	1	1	-	-	-	-	-	-	11
ACAT/ATGT	6	5	4	-	1	-	-	-	-	-	-	16
ACTC/AGTG	24	12	2	1	-	-	-	-	-	-	-	39
AGAT/ATCT	3	3	3	1	-	-	-	-	-	-	-	10
AGGG/CCCT	7	1	4	-	-	-	-	-	-	-	-	12
Other NNNN	37	17	7	3	-	-	-	-	-	-	-	64
												
NN	n. d.	868	395	210	160	116	76	59	50	53	108	2095
NNN	738	283	137	81	48	12	10	1	2	2	1	1315
NNNN	150	75	32	9	5	-	1	-	1	-	-	273
NNNNN	32	7	-	-	-	-	-	-	-	-	-	39
NNNNNN	1	-	-	-	-	-	-	-	-	-	-	1

n. d. = no data

**Table 2:** Accession designation, year of collection, origin, region and collector of 21 *Physcomitrella patens *accessions and two related *Funariaceae*.

Accession	Year	Origin	Region	Collector
Gransden (the worldwide labstrain)	1962	Great Gransden, Cambridgeshire, United Kingdom	Europe	H. L. K. Whithouse
NeneWashes	2003	Nene Washes, Cambridgeshire, United Kingdom	Europe	C. Preston
Cholsey	2001	Cholsey, Berkshire, United Kingdom	Europe	R. D. Porley
Gemünd-K1,	2002	Gemünd, Nordrhein-Westfalen, Germany	Europe	J.-P. Frahm
Gemünd-K5	2002	Gemünd, Nordrhein-Westfalen, Germany	Europe	J.-P. Frahm
Honnef	2003	Bad Honnef, Rheinland-Pfalz, Germany	Europe	J.-P. Frahm
Heimerbrühl	2003	Heimerbrühl, Rheinland-Pfalz, Germany	Europe	S. Caspari
Nennig	2003	Nennig, Saarland, Germany	Europe	S. Caspari
Martinshof	2003	Martinshof, Saarland, Germany	Europe	P. Wolff
Villersexel-K3	2003	Villersexel, Haute Saône, France	Europe	M. Lueth
Villersexel-K4	2003	Villersexel, Haute Saône, France	Europe	M. Lueth
Lviv	2003	Lviv, Ukraine	Europe	O. Lobachevska
Uppsala	2004	Wik castle, Uppsala, Sweden	Europe	A. Nilsson et al.
Trondheim	2002	Trondheim, Norway	Europe	K. Hassel
Kaskaskia	2003	Kaskaskia Island, Illinois, USA	Northern America	M. Sargent & D. Vitt
DelValleLake	2004	Del Valle Lake, California, USA	Northern America	B. D. Mishler
Okayama	1999	Okayama, Honshu, Japan	Japan	M. Hasebe
Kumamoto	1985	Kumamoto, Kyushu, Japan	Japan	K. Ono & H. Deguchi
Saitama	2002	Saitama, Honshu, Japan	Japan	M. Higuchi
Victoria	2003	Melton Reservoir, Victoria, Australia	Australia	V. Stajsic & N. Klazenga
Bisoke	2003	Mt. Bisoke, Ruhengeri, Rwanda	Africa	A. Solga & V. Buchbender
*Funaria hygrometrica*	n. a.	n. a., Germany	Europe	n. a.
*Physcomitrium sphaericum*	2001	Grosshartmannsdorf, Sachsen, Germany	Europe	J.-P. Frahm

n. a. = data not available
