# Supplementary material for: Identification of genic moss SSR markers and a comparative analysis of twenty-four algal and plant gene indices reveal species-specific rather than group-specific characteristics of microsatellites
Source: BMC Plant Biol. 2006 May 30;6:9. doi: 10.1186/1471-2229-6-9 (PMC1526434; doi:10.1186/1471-2229-6-9)
Supplement: Additional file 5 — PDF file with a table of the analysed plant gene indices. Tabular summary of the analysed plant gene indices including plant species name, taxonomic clade, source of database, release date, release version if specified in brackets, total number of sequences, total size of the databases in basepairs, average sequence length in basepairs and the GC-content of the databases. [file 1471-2229-6-9-S5.pdf]

Additional file 5

Tabular summary of the analysed plant gene indices including plant species name, taxonomic clade, source of database, release date, release version if specified in brackets, total number of sequences, total size of the databases in basepairs, average sequence length in basepairs and the GC-content of the databases.

Species	Taxonomic clade	Database	Total number of sequences	Total size in basebairs	Average sequence length in basepairs	GC-content in percentage
Chlamydomonas reinhardtii	Green alga	TIGR, Release 09.04 (5.0)	31,608	23,802,109	753	58.3
Mesostigma viride	Green alga	PlantGDB, Release 09.05 (149a)	5,467	3,752,103	686	51.7
Physcomitrella patens	Moss	COSMOSS, Release 03.04	48,961	37,672,030	769	46.9
Tortula ruralis	Moss	COSMOSS, Release 03.04	4,866	3,303,808	679	53.0
Adiantum capillus-veneris	Fern	PlantGDB, Release 09.05 (149a)	7,903	3,818,420	483	44.3
Cycas rumphii	Fern palm	NYPG, Release 09.05	4,210	2,003,527	476	46.8
Ginkgo biloba	Ginkgo	NYPG, Release 09.05	3,820	2,152,429	563	40.0
Pinus taeda	Conifer	TIGR, Release 07.05 (6.0)	45,557	36,548,862	802	43.8
Picea spec.	Conifer	TIGR, Release 09.04 (1.0)	27,194	18,454,828	679	41.6
Aquilegia formosa x *A. pubescens*	Dicot, Ranunculales	TIGR, Release 09.05 (2.0)	17,801	20,095,776	1,129	40.0
Mesembryanthemum crystallinum	Dicot, Caryophyllales	TIGR, Release 04.03 (3.0)	8,455	6,042,657	715	43.8
Beta vulgaris	Dicot, Caryophyllales	TIGR, Release 02.05 (1.2)	13,618	8,011,081	588	41.9
Vitis vinifera	Dicot, Eurosid incertae sedis	TIGR, Release 09.04 (4.0)	23,871	16,980,355	711	42.5
Medicago truncatula	Dicot, Fabales	TIGR, Release 01.05 (8.0)	36,878	28,175,361	764	39.4
Populus spec.	Dicot, Malpighiales	TIGR, Release 01.05 (2.0)	54,756	34,854,417	637	42.2
Arabidopsis thaliana	Dicot, Brassicales	TIGR, Release 08.04 (12.1)	42,301	50,086,382	1,184	42.4
Gossypium spec.	Dicot, Malvales	TIGR, Release 10.04 (6)	40,348	31,581,061	783	43.3
Solanum tuberosum	Dicot, Solanales	TIGR, Release 02.05 (10)	38,239	30,473,698	797	40.4
Helianthus annuus	Dicot, Asterales	TIGR, Release 08.03 (3.0)	20,520	9,811,686	478	42.6
Allium cepa	Monocot, Asparagales	TIGR, Release 07.04 (1)	11,726	8,741,722	745	41.7
Triticum aestivum	Monocot, Poales	TIGR, Release 02.05 (10)	122,282	79,823,548	653	51.1
Hordeum vulgare	Monocot, Poales	TIGR, Release 09.04 (9.0)	50,453	36,054,692	715	52.5
Saccharum officinarum	Monocot, Poales	TIGR, Release 10.04 (2.1)	78,547	59,153,866	753	51.5
Oryza sativa	Monocot, Poales	TIGR, Release 09.04 (16.0)	89,147	93,862,193	1,053	53.7

Significantly deviating values from the average calculated for average sequence length and GC-content are displayed in grey shaded cells.
