# Supplementary material for: Identification of genic moss SSR markers and a comparative analysis of twenty-four algal and plant gene indices reveal species-specific rather than group-specific characteristics of microsatellites
Source: BMC Plant Biol. 2006 May 30;6:9. doi: 10.1186/1471-2229-6-9 (PMC1526434; doi:10.1186/1471-2229-6-9)
Supplement: Additional file 1 — PDF file with the original data used to prepare the diagram in Figure 3. The additional file 1 contains the sizes of the analysed gene indices in bp, the total counts of detected SSRs, the total counts of dimer, trimer, tetramer, pentamer and hexamer SSRs as well as the calculated SSR counts per megabase for dimer, trimer, tetramer, pentamer and hexamer repeats. [file 1471-2229-6-9-S1.pdf]

	Chlamydomonas	*Mesostigma*	*Physcomitrella*	*Tortula*	*Adiantum*	*Cycas*	*Ginko*	*Pinus*	*Picea*	*Aquilegia*
Number of basepairs examined	23802109	3752103	37672030	3303808	3818420	2003527	2152429	36548862	18454828	20095776
No. of detected SSRs in total	2975	140	3723	375	268	106	135	1515	873	4822
No. of detected dimer SSRs	1647	16	2095	100	184	47	97	759	445	1483
No. of detected trimer SSRs	1229	59	1315	251	78	53	34	678	388	3050
No. of detected tetramer SSRs	69	53	273	17	5	6	2	25	16	184
No. of detected pentamer SSRs	10	11	39	1	0	0	0	10	10	42
No. of detected hexamer SSRs	20	1	1	6	1	0	2	43	14	63
Dimer counts/Mbp	69.2	4.3	55.6	30.3	48.2	23.5	45.1	20.8	24.1	73.8
Trimer counts/Mbp	51.6	15.7	34.9	76	20.4	26.5	15.8	18.6	21	151.8
Tetramer counts/Mbp	2.9	14.1	7.2	5.1	1.3	3	0.9	0.7	0.9	9.2
Pentamer counts/Mbp	0.4	2.9	1	0.3	0	0	0	0.3	0.5	2.1
Hexamer counts/Mbp	0.8	0.3	0	1.8	0.3	0	0.9	1.1	0.8	3.1
total SSRs counts/Mbp	125	37.3	98.8	113.5	70.2	52.9	62.7	41.5	47.3	240

Significantly deviating values from the average, calculated for the counts/Mbp, are displayed in grey shaded cells.

	Mesembryanthemum	*Beta*	*Vitis*	*Populus*	*Medicago*	*Arabidopsis*	*Gossypium*	*Solanum*	*Helianthus*	*Allium*
Number of basepairs examined	6042657	8011081	16980355	34854417	28175361	50086382	31581061	30473698	9811686	8741722
No. of detected SSRs in total	1444	1047	1864	5138	3186	5689	2711	2653	1263	340
No. of detected dimer SSRs	921	463	933	2584	1156	1760	1268	962	507	110
No. of detected trimer SSRs	458	523	812	2300	1860	3861	1273	1578	689	215
No. of detected tetramer SSRs	20	13	63	131	94	28	89	50	52	10
No. of detected pentamer SSRs	12	14	28	43	39	6	32	17	5	2
No. of detected hexamer SSRs	33	34	28	80	37	34	49	46	10	3
Dimer counts/Mbp	152.4	57.8	55	74.1	41	35.1	40.2	31.6	51.7	12.6
Trimer counts/Mbp	75.9	65.3	47.9	66	66	77.1	40.3	51.8	70.2	24.6
Tetramer counts/Mbp	3.3	1.6	3.7	3.8	3.3	0.6	2.8	1.6	5.3	1.1
Pentamer counts/Mbp	2	1.7	1.6	1.2	1.4	0.1	1	0.6	0.5	0.2
Hexamer counts/Mbp	5.5	4.2	1.6	2.3	1.3	0.7	1.6	1.5	1	0.3
total SSRs counts/Mbp	239	130.7	109.8	147.4	113.1	113.6	85.8	87.1	128.7	38.9

	Triticum	*Hordeum*	*Saccharum*	*Oryza*
Number of basepairs examined	79823548	36054692	59153866	93862193
No. of detected SSRs in total	10440	5098	7359	24247
No. of detected dimer SSRs	2996	1419	1937	4713
No. of detected trimer SSRs	6860	3297	4900	18939
No. of detected tetramer SSRs	415	273	193	384
No. of detected pentamer SSRs	114	59	238	106
No. of detected hexamer SSRs	55	50	91	105
Dimer counts/Mbp	37.5	39.4	32.7	50.2
Trimer counts/Mbp	85.9	91.4	82.8	201.8
Tetramer counts/Mbp	5.2	7.6	3.3	4.1
Pentamer counts/Mbp	1.4	1.6	4	1.1
Hexamer counts/Mbp	0.7	1.4	1.5	1.1
total SSRs counts/Mbp	130.8	141.3	124.4	258.3
