# Supplementary material for: Identification of genic moss SSR markers and a comparative analysis of twenty-four algal and plant gene indices reveal species-specific rather than group-specific characteristics of microsatellites
Source: BMC Plant Biol. 2006 May 30;6:9. doi: 10.1186/1471-2229-6-9 (PMC1526434; doi:10.1186/1471-2229-6-9)
Supplement: Additional file 2 — PDF file with the original data used to prepare the diagram in Figure 4. The additional file 2 contains the average length of dimer as well as trimer SSRs in the analysed 24 plant gene indices with the standard deviation in bp. [file 1471-2229-6-9-S2.pdf]

| | *Chlamydomonas* | *Mesostigma* | *Physcomitrella* | *Tortula* | *Adiantum* | *Cykas* | *Ginko* | *Pinus* | *Picea* | *Aquilegia* |
|---|---|---|---|---|---|---|---|---|---|---|
| Average SSR dimer length in basepairs | 18.61 | 13 | 16.68 | 15.8 | 16.95 | 14.63 | 19.43 | 24.47 | 23.39 | 19.87 |
| Standard deviation in basepairs | 14.15 | 1.46 | 8.76 | 5.41 | 6.38 | 7.19 | 10.85 | 20.67 | 17.41 | 10.21 |
| Average SSR trimer length in basepairs | 18.07 | 18.65 | 17.6 | 18.43 | 16.58 | 15.68 | 16.4 | 16.67 | 16.61 | 18.58 |
| Standard deviation in basepairs | 8.54 | 16.77 | 4.01 | 4.41 | 3.37 | 1.27 | 2.46 | 4.15 | 3.01 | 4.97 |

Significantly deviating values from the cumulative average are displayed in grey shaded cells.

| | *Mesembryanthemum* | *Beta* | *Vitis* | *Medicago* | *Populus* | *Arabidopsis* | *Gossypium* | *Solanum* | *Helianthus* | *Allium* |
|---|---|---|---|---|---|---|---|---|---|---|
| Average SSR dimer length in basepairs | 22.57 | 15.43 | 20.65 | 21.25 | 17.43 | 16.84 | 17.03 | 18.31 | 16.25 | 21.49 |
| Standard deviation in basepairs | 18.74 | 8.24 | 16.43 | 19.27 | 7.67 | 12.67 | 7.87 | 13.29 | 6.32 | 14.61 |
| Average SSR trimer length in basepairs | 18.5 | 17.54 | 18.29 | 17.94 | 18.14 | 17.84 | 18.59 | 16.95 | 16.99 | 17.13 |
| Standard deviation in basepairs | 4.85 | 4.38 | 5.13 | 4.59 | 4.72 | 13.2 | 6.2 | 3.75 | 3.84 | 3.75 |

| | *Triticum* | *Hordeum* | *Saccharum* | *Oryza* | Cumulative average |
|---|---|---|---|---|---|
| Average SSR dimer length in basepairs | 20.46 | 20.31 | 19.33 | 17.82 | 18.67 |
| Standard deviation in basepairs | 16.44 | 16.17 | 14.12 | 11.96 | |
| Average SSR trimer length in basepairs | 17.43 | 17.12 | 16.99 | 17.26 | 17.5 |
| Standard deviation in basepairs | 5.44 | 3.36 | 3.69 | 4.2 | |
