# Supplementary material for: Identification of genic moss SSR markers and a comparative analysis of twenty-four algal and plant gene indices reveal species-specific rather than group-specific characteristics of microsatellites
Source: BMC Plant Biol. 2006 May 30;6:9. doi: 10.1186/1471-2229-6-9 (PMC1526434; doi:10.1186/1471-2229-6-9)
Supplement: Additional file 3 — PDF file with the original data used to prepare the diagram in Figure 5. The additional file 3 contains the sizes of the analysed gene indices in bp, the total dimer SSR counts, the counts of the four canonical dimer SSR motifs as well as their calculated counts per megabase. [file 1471-2229-6-9-S3.pdf]

	Chlamydomonas	*Mesostigma*	*Physcomitrella*	*Tortula*	*Adiantum*	*Cycas*	*Ginko*
No. of basepairs examined	23802109	3752103	37672030	3303808	3818420	2003527	2152429
No. of SSRs consisting of the motif AC/GT	817	4	863	33	61	3	13
No. of SSRs consisting of the motif AG/CT	531	0	903	62	121	28	28
No. of SSRs consisting of the motif AT/TA	112	7	307	0	0	16	56
No. of SSRs consisting of the motif CG/GC	187	5	22	5	2	0	0
Total no. of detected dimer SSRs	1647	16	2095	100	184	47	97
counts/Mbp of SSRs consisting of the motif AC/GT	34,3	1,1	22,9	10	16	1,5	6
counts/Mbp of SSRs consisting of the motif AG/CT	22,3	0	24	20	31,7	14	13
counts/Mbp of SSRs consisting of the motif AT/TA	4,7	1,9	8,2	0	0	8	26
counts/Mbp of SSRs consisting of the motif CG/GC	7,9	1,3	0,6	1,7	0,5	0	0
Total dimer SSR counts/Mbp	69,2	4,3	55,6	30,3	48,2	23,5	45,1

Significantly deviating values from the average, calculated for the counts/Mbp, are displayed in grey shaded cells.

	Pinus	*Picea*	*Aquilegia*	*Mesembryanthemum*	*Beta*	*Vitis*	*Populus*
No. of basepairs examined	36548862	18454828	20095776	6042657	8011081	16980355	34854417
No. of SSRs consisting of the motif AC/GT	52	34	85	33	42	55	336
No. of SSRs consisting of the motif AG/CT	228	101	1219	833	364	561	1559
No. of SSRs consisting of the motif AT/TA	472	308	178	49	54	316	665
No. of SSRs consisting of the motif CG/GC	7	2	1	6	3	1	24
Total no. of detected dimer SSRs	759	445	1483	921	463	933	2584
counts/Mbp of SSRs consisting of the motif AC/GT	1,4	1,8	4,2	5,5	5,2	3,2	9,6
counts/Mbp of SSRs consisting of the motif AG/CT	6,2	5,5	60,7	137,9	45,6	33	44,7
counts/Mbp of SSRs consisting of the motif AT/TA	12,9	16,7	8,9	8,1	6,7	18,6	19,1
counts/Mbp of SSRs consisting of the motif CG/GC	0,2	0,1	0,05	1	0,4	0,1	0,7
Total dimer SSR counts/Mbp	20,8	24,1	73,8	152,4	57,8	55	74,1

	Medicago	*Arabidopsis*	*Gossypium*	*Solanum*	*Helianthus*	*Allium*	*Triticum*	*Hordeum*
No. of basepairs examined	28175361	50086382	31581061	30473698	9811686	8741722	79823548	36054692
No. of SSRs consisting of the motif AC/GT	143	173	145	123	108	32	782	353
No. of SSRs consisting of the motif AG/CT	658	1347	437	329	300	19	1683	781
No. of SSRs consisting of the motif AT/TA	331	235	652	509	97	58	338	189
No. of SSRs consisting of the motif CG/GC	24	5	34	1	2	1	193	96
Total no. of detected dimer SSRs	1156	1760	1268	962	507	110	2996	1419
counts/Mbp of SSRs consisting of the motif AC/GT	5,1	3,5	4,6	4	11	3,7	9,8	9,8
counts/Mbp of SSRs consisting of the motif AG/CT	23,4	26,9	13,8	10,8	30,6	2,2	21,1	21,7
counts/Mbp of SSRs consisting of the motif AT/TA	11,7	4,7	20,6	16,7	9,9	8,6	4,2	5,2
counts/Mbp of SSRs consisting of the motif CG/GC	0,9	1	1,1	0	0,2	0	2,4	2,7
Total dimer SSR counts/Mbp	41	35,1	40,2	31,6	51,7	12,6	37,5	39,4

	Saccharum	*Oryza*
No. of basepairs examined	59153866	93862193
No. of SSRs consisting of the motif AC/GT	354	630
No. of SSRs consisting of the motif AG/CT	986	3008
No. of SSRs consisting of the motif AT/TA	425	523
No. of SSRs consisting of the motif CG/GC	172	552
Total no. of detected dimer SSRs	1937	4713
counts/Mbp of SSRs consisting of the motif AC/GT	6	6,7
counts/Mbp of SSRs consisting of the motif AG/CT	16,7	32
counts/Mbp of SSRs consisting of the motif AT/TA	7,2	5,6
counts/Mbp of SSRs consisting of the motif CG/GC	2,9	5,9
Total dimer SSR counts/Mbp	32,7	50,2
