# Supplementary material for: Identification of genic moss SSR markers and a comparative analysis of twenty-four algal and plant gene indices reveal species-specific rather than group-specific characteristics of microsatellites
Source: BMC Plant Biol. 2006 May 30;6:9. doi: 10.1186/1471-2229-6-9 (PMC1526434; doi:10.1186/1471-2229-6-9)
Supplement: Additional file 4 — PDF file with the original data used to prepare the diagram in Figure 6. The additional file 4 contains the sizes of the analysed gene indices in bp, the total trimer SSR counts, the counts of the 10 canonical dimer SSR motifs as well as their calculated counts per megabase. [file 1471-2229-6-9-S4.pdf]

	Chlamydomonas	*Mesostigma*	*Physcomitrella*	*Tortula*	*Adiantum*	*Cycas*	*Ginko*	*Pinus*
No. of basepairs examined	23802109	3752103	37672030	3303808	3818420	2003527	2152429	36548862
No. of SSRs consisting of the motif AAC/GTT	21	1	138	11	3	0	2	51
No. of SSRs consisting of the motif AAG/CTT	28	22	229	21	10	22	7	130
No. of SSRs consisting of the motif AAT/ATT	5	4	102	1	0	7	9	93
No. of SSRs consisting of the motif ACC/GGT	89	0	123	17	15	2	5	54
No. of SSRs consisting of the motif ACG/CGT	120	5	160	45	5	0	0	42
No. of SSRs consisting of the motif ACT/ATG	6	1	41	8	21	1	3	30
No. of SSRs consisting of the motif AGC/GCT	354	6	211	104	5	12	2	113
No. of SSRs consisting of the motif AGG/CCT	66	13	228	14	4	7	3	98
No. of SSRs consisting of the motif AGT/ACT	9	2	71	12	13	2	3	24
No. of SSRs consisting of the motif CCG/CGG	531	5	12	18	2	0	0	43
Total no. of detected trimer SSRs	1229	59	1315	251	78	53	34	678
counts/Mbp of SSRs consisting of the motif AAC/GTT	0,9	0,3	3,7	3,3	0,8	0	0,9	1,4
counts/Mbp of SSRs consisting of the motif AAG/CTT	1,2	5,9	6,1	6,4	2,6	11	3,3	3,6
counts/Mbp of SSRs consisting of the motif AAT/ATT	0,2	1,1	2,7	0,3	0	3,5	4,2	2,5
counts/Mbp of SSRs consisting of the motif ACC/GGT	3,7	0	3,3	5,1	3,9	1	2,3	1,5
counts/Mbp of SSRs consisting of the motif ACG/CGT	5	1,3	4,2	13,6	1,3	0	0	1,1
counts/Mbp of SSRs consisting of the motif ACT/ATG	0,3	0,3	1,1	2,4	5,5	0,5	1,4	0,8
counts/Mbp of SSRs consisting of the motif AGC/GCT	14,9	1,6	5,6	31,5	1,3	6	0,9	3,1
counts/Mbp of SSRs consisting of the motif AGG/CCT	2,8	3,5	6,1	4,2	1	3,5	1,4	2,7
counts/Mbp of SSRs consisting of the motif AGT/ACT	0,4	0,5	1,9	3,6	3,4	1	1,4	0,7
counts/Mbp of SSRs consisting of the motif CCG/CGG	22,3	1,3	0,3	5,4	0,5	0	0	1,2
Total trimer SSR counts/Mbp	51,6	15,7	34,9	76	20,4	26,5	15,8	18,6

Significantly deviating values from the average, calculated for the counts/Mbp, are displayed in grey shaded cells.

	Picea	*Aquilegia*	*Mesembryanthemum*	*Beta*	*Vitis*	*Populus*	*Medicago*	*Arabidopsis*
No. of basepairs examined	18454828	20095776	6042657	8011081	16980355	34854417	28175361	50086382
No. of SSRs consisting of the motif AAC/GTT	15	386	67	113	30	134	231	434
No. of SSRs consisting of the motif AAG/CTT	68	1477	98	87	231	550	655	1768
No. of SSRs consisting of the motif AAT/ATT	83	142	26	41	125	242	206	45
No. of SSRs consisting of the motif ACC/GGT	18	292	69	62	97	304	162	237
No. of SSRs consisting of the motif ACG/CGT	24	62	21	20	37	198	67	76
No. of SSRs consisting of the motif ACT/ATG	19	254	49	72	65	135	170	463
No. of SSRs consisting of the motif AGC/GCT	40	98	29	25	95	260	67	121
No. of SSRs consisting of the motif AGG/CCT	86	76	29	24	70	277	96	361
No. of SSRs consisting of the motif AGT/ACT	13	261	58	68	41	124	193	323
No. of SSRs consisting of the motif CCG/CGG	22	2	12	11	21	76	13	33
Total no. of detected trimer SSRs	388	3050	458	523	812	2300	1860	3861
counts/Mbp of SSRs consisting of the motif AAC/GTT	0,8	19,2	11,1	14,1	8,7	8,2	3,8	1,8
counts/Mbp of SSRs consisting of the motif AAG/CTT	3,7	73,5	16,2	10,9	35,3	23,2	15,8	13,6
counts/Mbp of SSRs consisting of the motif AAT/ATT	4,5	7,1	4,3	5,1	0,9	7,3	6,9	7,4
counts/Mbp of SSRs consisting of the motif ACC/GGT	1	14,5	11,4	7,7	4,7	5,8	8,7	5,7
counts/Mbp of SSRs consisting of the motif ACG/CGT	1,3	3,1	3,5	2,5	1,5	2,4	5,7	2,2
counts/Mbp of SSRs consisting of the motif ACT/ATG	1	12,6	8,1	9	9,2	6	3,9	3,8
counts/Mbp of SSRs consisting of the motif AGC/GCT	2,2	4,9	4,8	3,1	2,4	2,4	7,5	5,6
counts/Mbp of SSRs consisting of the motif AGG/CCT	4,7	3,8	4,8	3	7,2	3,4	7,9	4,1
counts/Mbp of SSRs consisting of the motif AGT/ACT	0,7	13	9,6	8,5	6,4	6,9	3,6	2,4
counts/Mbp of SSRs consisting of the motif CCG/CGG	1,2	0,1	2	1,4	0,7	0,5	2,2	1,2
Total trimer SSR counts/Mbp	21	151,8	75,9	65,3	47,9	66	66	77,1

	Gossypium	*Solanum*	*Helianthus*	*Allium*	*Triticum*	*Hordeum*	*Saccharum*	*Oryza*
No. of basepairs examined	31581061	30473698	9811686	8741722	79823548	36054692	59153866	93862193
No. of SSRs consisting of the motif AAC/GTT	102	190	40	12	468	83	137	165
No. of SSRs consisting of the motif AAG/CTT	341	455	105	47	502	266	190	891
No. of SSRs consisting of the motif AAT/ATT	158	201	90	16	73	34	50	110
No. of SSRs consisting of the motif ACC/GGT	139	181	188	13	427	202	332	1113
No. of SSRs consisting of the motif ACG/CGT	62	52	33	12	523	269	477	1474
No. of SSRs consisting of the motif ACT/ATG	120	132	75	18	206	110	122	311
No. of SSRs consisting of the motif AGC/GCT	86	102	50	38	920	468	692	2003
No. of SSRs consisting of the motif AGG/CCT	66	92	29	33	1041	519	615	2830
No. of SSRs consisting of the motif AGT/ACT	144	96	49	16	128	67	51	170
No. of SSRs consisting of the motif CCG/CGG	55	76	30	10	2572	1279	2234	9872
Total no. of detected trimer SSRs	1273	1578	689	215	6860	3297	4900	18939
counts/Mbp of SSRs consisting of the motif AAC/GTT	3,22	6,2	4,1	1,4	5,9	2,3	2,3	1,8
counts/Mbp of SSRs consisting of the motif AAG/CTT	10,8	14,9	10,7	5,4	6,3	7,4	3,2	9,5
counts/Mbp of SSRs consisting of the motif AAT/ATT	5	6,6	9,2	1,8	0,9	0,9	0,8	1,2
counts/Mbp of SSRs consisting of the motif ACC/GGT	4,4	5,9	19,22	1,5	5,3	5,6	5,6	11,9
counts/Mbp of SSRs consisting of the motif ACG/CGT	2	1,7	3,4	1,4	6,6	7,5	8,1	15,7
counts/Mbp of SSRs consisting of the motif ACT/ATG	3,8	4,3	7,6	2,1	2,6	3,1	2,1	3,3
counts/Mbp of SSRs consisting of the motif AGC/GCT	2,7	3,3	5,1	4,3	11,5	13	11,7	21,3
counts/Mbp of SSRs consisting of the motif AGG/CCT	2,1	3	3	3,8	13	14,4	10,4	30,2
counts/Mbp of SSRs consisting of the motif AGT/ACT	4,6	3,2	5	1,8	11,6	1,9	0,9	1,8
counts/Mbp of SSRs consisting of the motif CCG/CGG	1,5	2,5	3,1	1,1	32,2	35,5	37,8	105,2
Total trimer SSR counts/Mbp	40,3	51,8	70,2	24,6	85,9	91,4	82,8	201,8
